# Role of Eosinophils and Tumor Necrosis Factor Alpha in Interleukin-25-Mediated Protection from Amebic Colitis

**DOI:** 10.1128/mBio.02329-16

**Published:** 2017-02-28

**Authors:** Zannatun Noor, Koji Watanabe, Mayuresh M. Abhyankar, Stacey L. Burgess, Erica L. Buonomo, Carrie A. Cowardin, William A. Petri

**Affiliations:** a Division of Infectious Diseases and International Health, Department of Medicine, University of Virginia Health System, Charlottesville, Virginia, USA; b AIDS Clinical Center, National Center for Global Health and Medicine, Tokyo, Japan; National Institute of Allergy and Infectious Diseases

## Abstract

The parasite *Entamoeba histolytica* is a cause of diarrhea in infants in low-income countries. Previously, it was shown that tumor necrosis factor alpha (TNF-α) production was associated with increased risk of *E. histolytica* diarrhea in children. Interleukin-25 (IL-25) is a cytokine that is produced by intestinal epithelial cells that has a role in maintenance of gut barrier function and inhibition of TNF-α production. IL-25 expression was decreased in humans and in the mouse model of amebic colitis. Repletion of IL-25 blocked *E. histolytica* infection and barrier disruption in mice, increased gut eosinophils, and suppressed colonic TNF-α. Depletion of eosinophils with anti-Siglec-F antibody prevented IL-25-mediated protection. In contrast, depletion of TNF-α resulted in resistance to amebic infection. We concluded that IL-25 provides protection from amebiasis, which is dependent upon intestinal eosinophils and suppression of TNF-α.

## IMPORTANCE

The intestinal epithelial barrier is important for protection from intestinal amebiasis. We discovered that the intestinal epithelial cytokine IL-25 was suppressed during amebic colitis in humans and that protection could be restored in the mouse model by IL-25 administration. IL-25 acted via eosinophils and suppressed TNF-α. This work illustrates a previously unrecognized pathway of innate mucosal immune response.

## INTRODUCTION

One of the leading causes of death in children under 5 years of age globally is diarrheal disease. The intestinal parasite *Entamoeba histolytica* is a common cause of severe diarrhea (1), as recently reaffirmed by two large multicenter studies (2, 3). While most infections are asymptomatic, up to 20% lead to diarrhea (4). The varied outcomes of *E. histolytica* infection are likely due to a combination of parasite, host, and environmental factors (5).

*E. histolytica* disrupts the mucosal barrier in a sequential process of adherence to intestinal epithelial cells by a parasite Gal/GalNAc lectin, followed by killing of the epithelial cells in a nibbling process termed amebic trogocytosis, leading to penetration of the epithelium and destruction of underlying tissue (6–8). *E. histolytica* induces several proinflammatory cytokines, including interleukin-1β (IL-1β), IL-23, IL-17, and tumor necrosis factor alpha (TNF-α) (9–11). One of the mechanisms that lead to cytokine production is activation of the inflammasome (12–14). While the inflammatory response represents a line of defense, an excessive response may contribute to the tissue damage seen in amebic colitis (15). High TNF-α correlated with diarrhea and disease severity in children infected with *E. histolytica*, and blocking TNF-α with neutralizing monoclonal antibody (MAb) was shown to reduce inflammation and intestinal damage (16–18). TNF-α induces macrophages and neutrophils to produce reactive oxygen species and nitric oxide to kill *E. histolytica*; however, oxygen free radicals may also be responsible for collateral tissue damage (15).

Epithelial tuft cells in the bowel produce the cytokine IL-25, which has multiple functions (19). TNF-α negatively regulates IL-25 production in the human gut (20). IL-25 can activate both innate and adaptive sources to induce type 2 cytokines, such as IL-4, IL-5, IL-9, and IL-13 (21). Commensal gut bacteria increase IL-25 production by epithelial intestinal cells (22). IL-25 is also a potent inducer of the antimicrobial peptide angiogenin-4 in an IL-13-dependent manner (23). IL-25 induces eosinophil infiltration in the gut (24), which is potentially relevant in amebiasis as eosinophilia was also associated with reduced size and number of amebic liver abscesses in the gerbil model (25). However, the role of IL-25 in amebic colitis remains unknown.

Here, we demonstrate that the anti-inflammatory cytokine IL-25 is suppressed during *E. histolytica* infection in humans and in a mouse model of amebic colitis. We tested the role of IL-25 by administering recombinant IL-25 (rIL-25) to mice during amebic colitis and discovered that IL-25 protected against amebic colitis and that this protection was eosinophil dependent and acted in part by suppressing TNF-α.

## RESULTS

### IL-25 is suppressed during *E. histolytica* infection in humans and in the mouse model.

**FIG 1:**
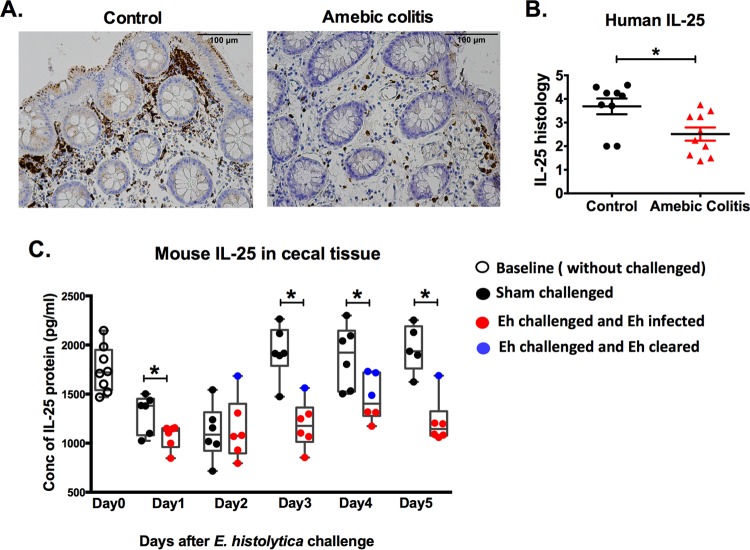
IL-25 is suppressed during *E. histolytica* infection in humans and mouse model. (A) Representative IL-25 immunohistochemical staining of human biopsy samples taken from the colon of control and amebic colitis patients. (B) Histological scoring for IL-25 in the human colon biopsy specimens. Control patients included patients with diarrhea, polyps, and Crohn’s disease. (C) IL-25 protein levels were measured in cecal tissue lysates of mice before (day 0) and after *E. histolytica* cecal challenge. *, *P* < 0.05.

Because of the importance of the microbiome and the intestinal epithelium in defense against *E. histolytica* colitis (26–28), we hypothesized that IL-25 would be protective against amebic infection in the cecum. Human colon biopsy samples from controls (from the University of Virginia) and amebic colitis patients were stained for IL-25 by immunohistochemistry. IL-25 was less abundant in amebic colitis patients (Fig. 1A and B).

We utilized the mouse model of amebic colitis to test the importance of IL-25 in defense against *E. histolytica* invasion. CBA/J mice were challenged with *E. histolytica* and compared to control mice that received a sham challenge. IL-25 was decreased at day 1 and day 2 in both groups but remained depressed after day 3 only in the *E. histolytica*-challenged mice. Sham-challenged mice returned to baseline levels of IL-25 after day 3 (Fig. 1C). Mice that cleared *E. histolytica* infection had a non-statistically significant higher level of IL-25 (Fig. 1C, solid blue circles) than mice that remained infected (solid red circles). We concluded that IL-25 was reduced with *E. histolytica* infection both in humans and in the mouse model.

### IL-25 had a protective role against *E. histolytica* colitis in the mouse model.

**FIG 2:**
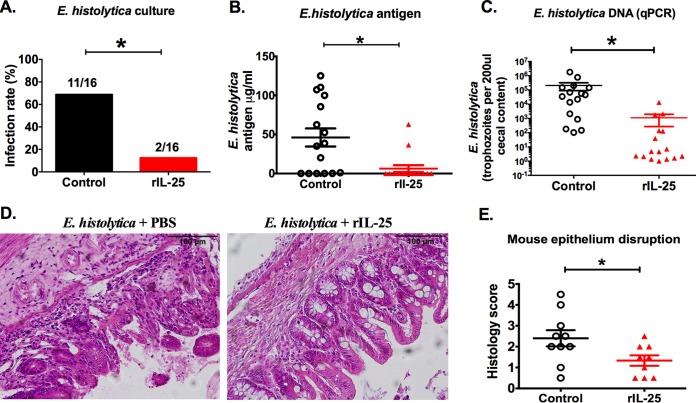
IL-25 prevents *E. histolytica* infection and development of colitis in the mouse model. (A to C) Mice were injected intraperitoneally with 0.5 µg recombinant IL-25 (red bar or solid red triangle) or PBS (black bar or open circle) each day for 8 days and were challenged with *E. histolytica* on day 5. Cecal contents were harvested 7 days after *E. histolytica* challenge (day 12), and parasite infection was evaluated by culture (A), *E. histolytica* antigen detection (B), and *E. histolytica* DNA (C). (D) Representative cecal histopathology of *E. histolytica*-challenged mice with or without IL-25 treatment. (E) Epithelial disruption score in *E. histolytica* challenged with or without rIL-25. *, *P* < 0.05.

In order to test whether IL-25 would protect mice against amebiasis, we injected into the peritoneum 0.5 µg of rIL-25 or phosphate-buffered saline (PBS) daily for 4 days prior to and 4 days after *E. histolytica* challenge. *E. histolytica* infection and parasite burden in the cecum were decreased in the rIL-25-treated group (Fig. 2A to C). Epithelial disruption was reduced in the rIL-25-treated group at 7 days after *E. histolytica* challenge (Fig. 2D and E). We concluded that IL-25 reduced *E. histolytica* burden and maintained the gut barrier.

### rIL-25 administration induced type 2 responses.

**FIG 3:**
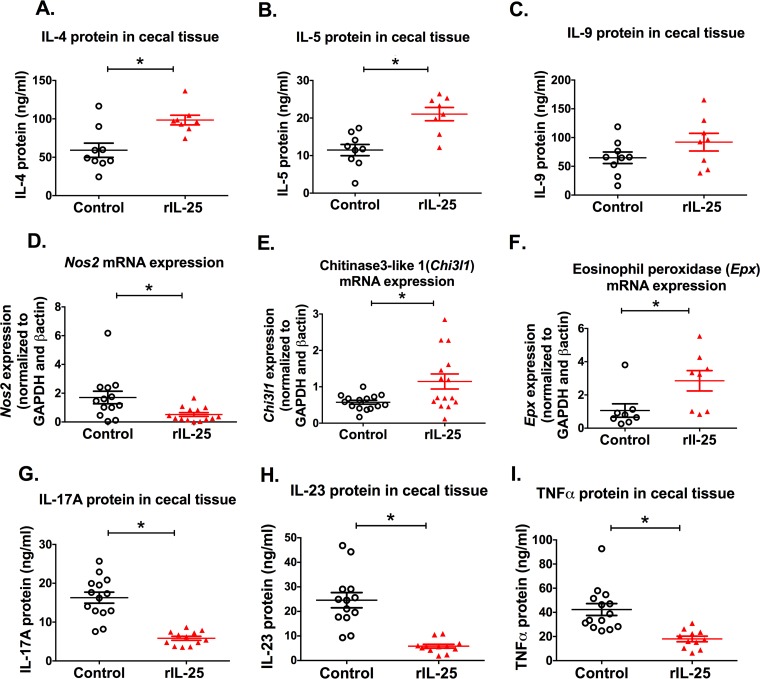
rIL-25 administration increased type 2 responses and suppressed inflammatory responses in *E. histolytica*-challenged mice. Cecal tissue was collected from rIL-25- or PBS-treated mice 7 days after *E. histolytica* challenge, and the cytokines IL-4, IL-5, IL-9, IL-17, IL-23, and TNF-α were measured by ELISA (A to C and G to I). Cytokine concentrations were normalized to total protein concentration. Inducible nitric oxide synthase (*Nos2*), chitinase 3-like 1 (*Chi3l1*), and eosinophil peroxidase (*Epx*) mRNAs were measured from cecal tissue of *E. histolytica*-challenged mice with or without rIL-25 treatment (D to F) after 1 day of *E. histolytica* challenge. *, *P* < 0.05.

IL-25 regulates type 2 immunity in the gut via type 2 innate lymphoid cells (21). In order to describe the immune response induced by IL-25 during amebiasis, we measured IL-4, IL-5, and IL-9 by enzyme-linked immunosorbent assay (ELISA) from cecal tissue lysates of mice after *E. histolytica* challenge with or without rIL-25 treatment. We found that IL-4 and IL-5 were elevated in rIL-25-treated mice (Fig. 3A and B), whereas IL-9 was not (Fig. 3C). Inducible nitric oxide synthase-encoding mRNA (*Nos2*) was decreased in the presence of rIL-25 (Fig. 3D). On the other hand, the amount of mRNA encoding chitinase 3-like 1 (*Chi3l1*) and eosinophil peroxidase (*Epx*) was upregulated in the presence of rIL-25 (Fig. 3E and F). These data suggested that rIL-25 induces alternatively activated macrophages during *E. histolytica* infection. We also measured inflammatory cytokines known to be suppressed by IL-25: IL-23, IL-17, and TNF-α. These cytokines as expected were decreased in the cecal tissue lysate of rIL-25-treated mice (Fig. 3G to I).

### Eosinophils were important for IL-25-mediated protection.

**FIG 4:**
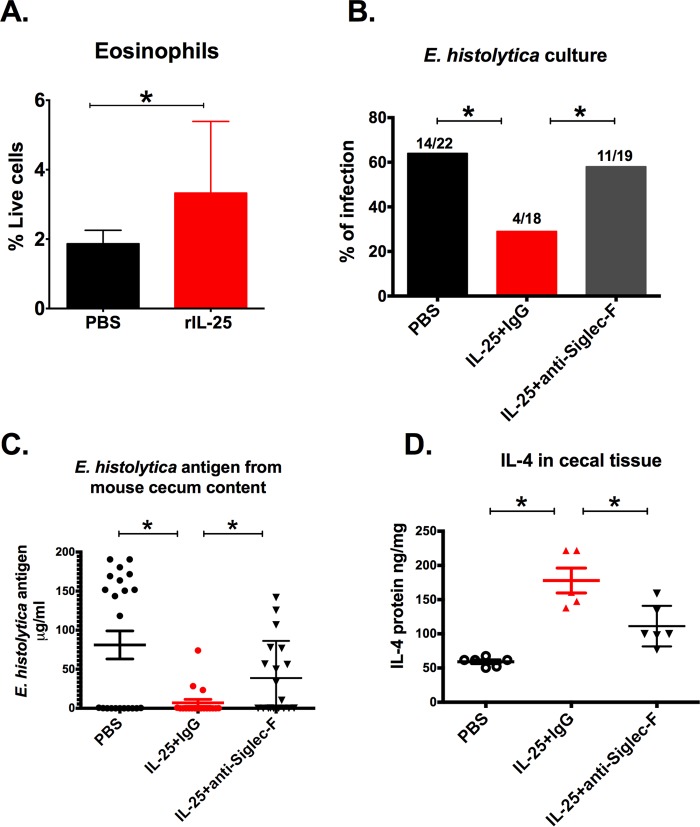
IL-25-dependent protection against amebiasis was eosinophil dependent. Mice were injected intraperitoneally with 0.5 µg recombinant IL-25 daily for 4 days prior to the challenge and harvested 24 h postchallenge. (A) Cecal lamina propria cells were isolated, and CD45^+^ CD11b^+^ CD11c^mid^ Siglec-F^+^ Ly6G^−^ eosinophils were quantified as a percentage of live cells by flow cytometry. (B to D) Forty micrograms of anti-Siglec-F or an isotype control MAb was administered intraperitoneally from 1 day prior to *E. histolytica* challenge to 3 days after challenge (3 doses). Mice were euthanized 7 days after *E. histolytica* challenge (day 12), and cecal contents were harvested to evaluate infection by culture (B) and *E. histolytica* antigen detection (C). (D) IL-4 protein was measured from cecal tissue lysate with or without eosinophil depletion. *, *P* < 0.05.

IL-25 is known to increase eosinophils in the gut, and IL-25-induced eosinophilia protects against pathogenesis in *Clostridium difficile* colitis (24). Increased gut eosinophil peroxidase (*Epx*) mRNA with IL-25 suggested that eosinophils could be important for protection against amebic colitis (Fig. 3F). Increased eosinophils in the lamina propria during amebic infection were detected by flow cytometry (Fig. 4A). We tested the importance of eosinophils in protection against amebic colitis by depletion with anti-Siglec-F. Anti-Siglec-F monoclonal antibody or an IgG2a isotype control antibody was administered to IL-25-treated mice. Depletion of eosinophils abrogated IL-25 protection (Fig. 4B and C) and decreased gut IL-4 (Fig. 4D), as expected since eosinophils are a major source of IL-4 (29). These findings indicated that eosinophils were required for IL-25-mediated protection.

### IL-25-mediated protection was accompanied by decreased TNF-α in the mouse model.

**FIG 5:**
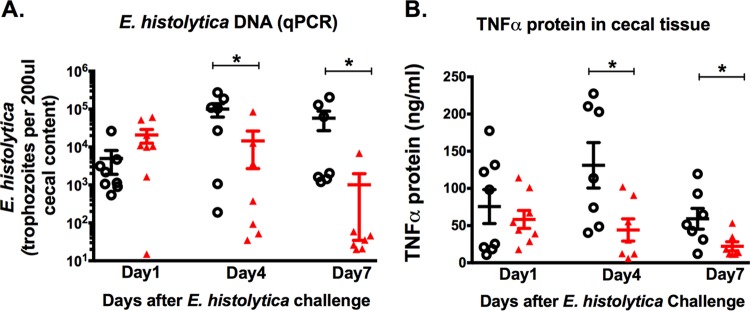
IL-25-mediated protection against amebic colitis was associated with suppression of TNF-α in the cecum in the mouse model. Mice were injected intraperitoneally with 0.5 µg recombinant IL-25 (solid red triangle) or PBS (open black circle) each day for 4 days prior to infection and 4 days after infection. Mice were euthanized 1, 4, or 7 days after *E. histolytica* challenge. (A) *E. histolytica* DNA detection from cecal contents. (B) TNF-α measured from cecal tissue lysate. *, *P* < 0.05.

rIL-25-treated mice contained lower levels of *E. histolytica* on days 4 and 7 after *E. histolytica* challenge (Fig. 5A). TNF-α was decreased on both day 4 and day 7 in the presence of rIL-25 (Fig. 5B).

### TNF-α is upregulated during amebic colitis in humans, and anti-TNF-α treatment protected mice from amebiasis.

**FIG 6:**
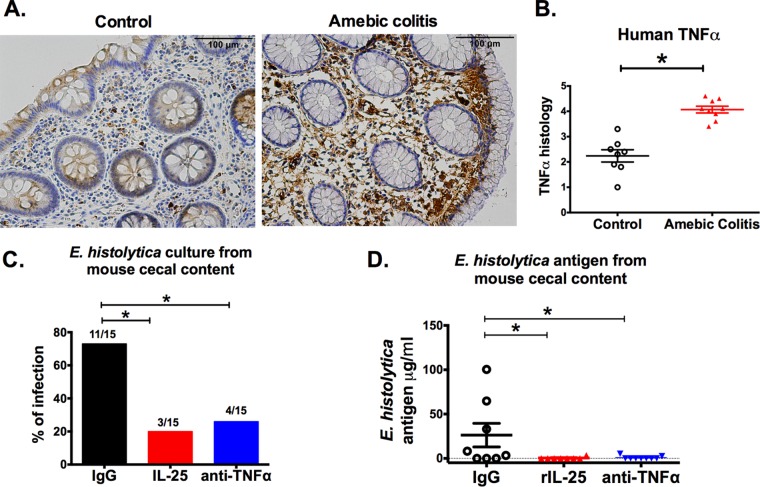
TNF-α increased during *E. histolytica* infection in humans, and anti-TNF-α treatment protected mice from amebiasis. (A) Representative photomicrographs of TNF-α immunohistochemical staining from human biopsy samples taken from the colon of control and amebic colitis patients. (B) Histological scoring for TNF-α in human colon biopsy specimens. Control patients included patients with diarrhea, polyps, and Crohn’s disease. (C and D) Mice were injected intraperitoneally with an IgG1 isotype control antibody, 0.5 µg recombinant IL-25 daily, or 0.5 μg anti-TNF-α intraperitoneally on alternate days from 1 day prior to *E. histolytica* challenge to 3 days after challenge (3 doses). Mice were euthanized 7 days after *E. histolytica* challenge. (C) *E. histolytica* culture positivity. (D) *E. histolytica* antigen in cecal content. *, *P* < 0.05.

TNF-α was measured in colon biopsy samples collected from amebic colitis and control patients by immunohistochemistry (Fig. 6A). TNF-α protein was higher in amebic colitis patients as assessed by immunohistochemistry (Fig. 6B). From these results, we hypothesized that suppression of TNF-α expression was a possible mechanism by which IL-25 mediates protection against amebiasis. To test this, TNF-α was neutralized by use of a monoclonal antibody in CBA/J mice. There was a lower infection rate (Fig. 6C) and lower antigen load (Fig. 6D) in mice treated with anti-TNF-α antibody. IL-25 suppression of TNF-α could be one potential mechanism of the protective action of IL-25.

## DISCUSSION

The most important finding of our study is that the intestinal epithelial cytokine IL-25 protects against amebic colitis. IL-25 was suppressed in the colon of humans with amebic colitis and in the murine model, and repletion of IL-25 reduced *E. histolytica* infection and protected the gut epithelial barrier.

IL-25 provided protection from amebic colitis in an eosinophil-dependent process, as demonstrated by abrogation of protection by depletion of eosinophils with anti-Siglec-F. Siglec-F is a cell surface lectin belonging to the Ig superfamily that binds glycol conjugates containing sialic acids that are commonly found on eosinophils in mice, although it was also reported that various cell types, such as alveolar macrophages and intestinal M cells, express this surface protein (30, 31). Recently, Buonomo et al. have reported that IL-25 also is protective against *Clostridium difficile* infection through eosinophil induction (24). Additionally, *C. difficile* infection with strains containing CDT (*Clostridium difficile* transferase toxin) causes more severe pathology because they suppress the accumulation of eosinophils in the lamina propria of the colon (32). For *C. difficile* infection, IL-25 acted by reducing host inflammation but not pathogen burden, whereas for amebic colitis, both pathogen burden and inflammation were suppressed. Therefore, there may be differences in how eosinophils act to protect from amebiasis and *C. difficile*.

The role of eosinophils in amebiasis has not been studied intensively (33). Induction of eosinophils by use of *Toxocara canis* antigen resulted in reduced amebic liver abscess size and number in an animal model. Also supporting a role for eosinophils in amebiasis is the observation that degenerated eosinophil products (Charcot-Leiden crystals) are present along with trophozoites in the stool of patients with amebiasis (34).

We hypothesized that an IL-25-induced influx of eosinophils would ultimately function to suppress gut inflammation to provide protection against amebic colitis. We expected that IL-25-mediated protection in amebiasis would be associated with a shift from a proinflammatory response to type 2 immunity. In this context, we found that rIL-25 administration in mice in fact suppressed expression of inflammatory cytokines (IL-23, IL-17, and TNF-α) and induced type 2 cytokines (IL-4 and IL-5).

The proinflammatory cytokine TNF-α is known to play a crucial role in intestinal inflammation during amebic colitis (16). We reported here that TNF-α in human colon biopsy specimens was elevated during amebic colitis and that administration of rIL-25 decreased TNF-α in the mouse model during amebic colitis. Furthermore, TNF-α depletion rendered mice resistant to *E. histolytica* infection. This was of particular import, as amebic diarrhea is positively correlated with TNF-α production in humans (17). Considered together, TNF-α suppression by eosinophils is a possible mechanism of IL-25-mediated protection, although the detailed interaction between TNF-α and IL-25 has not been studied in the present study.

IL-25 induction of alternatively activated macrophages could be a mechanism of TNF-α suppression. Eosinophils are known as a major source of IL-4 that induces macrophage polarization into anti-inflammatory macrophages (35). In our study, we found that IL-25 induction of IL-4 was blocked when eosinophils were depleted with monoclonal antibodies. IL-25 administration caused downregulation of nitric oxide synthase 2 (*Nos2*) and upregulation of chitinase 3-like 1 (*Chi3l1*), a transcript present in type 2 alternatively activated macrophages.

Although it has not been studied whether IL-25 acts directly on eosinophils, direct stimulation by IL-25 may be possible as the IL-25 receptor IL-25RB has been reported to be expressed on human eosinophils (36). It also has not been investigated whether eosinophil-induced IL-4 can cause macrophage polarization to suppress TNF-α. Further investigation is required to understand the cellular and molecular mechanism of the IL-25–eosinophil pathway during amebiasis. The hypothesis that IL-25 acts to protect from amebiasis through eosinophil conversion of type 1 to type 2 macrophages therefore remains to be tested.

In conclusion, we have found that IL-25 is suppressed during amebic colitis and that administration of IL-25 in a mouse model reduced *E. histolytica* trophozoite number, antigen load, and epithelial disruption in the cecum. Moreover, we demonstrated that eosinophils induced by IL-25 may protect by suppressing TNF-α, as IL-25 suppressed TNF-α levels and neutralization of TNF-α prevented amebic colitis.

## MATERIALS AND METHODS

### Mice.

Six-week-old male CBA/J mice and C57BL/6J mice were purchased from the Jackson Laboratory (Bar Harbor, ME). Mice were housed in a specific-pathogen–free facility in microisolator cages and provided autoclaved food (lab diet 5010) and water *ad libitum*. The University of Virginia Institutional Animal Care and Use Committee approved all procedures.

### Recombinant IL-25 treatment.

Mice were injected intraperitoneally with 0.5 μg recombinant IL-25 (R&D Systems) or 100 μl PBS each day for 4 days before and through 4 days after *E. histolytica* challenge.

### *E. histolytica* infection evaluation.

Mice were intracecally challenged with 2 million trophozoites in 150 μl of medium using laparotomy (37). Mice were sacrificed 7 days after *E. histolytica* challenge, and cecal contents were collected to evaluate infection by culture, *E. histolytica* antigen detection, and *E. histolytica* DNA detection. Cecal contents were cultured in complete TYI-S-33 medium with supplemental antibiotics for 3 days at 37°C. *E. histolytica* antigen was detected in cecal content using the *E. histolytica* II ELISA kit (Techlab, Blacksburg, VA). Cecal contents were used for DNA isolation using the Qiagen DNA extraction kit (Qiagen, Hilden, Germany), and a quantitative real-time PCR (qPCR) assay was used for *E. histolytica* DNA measurement. For quantification, standards were generated by isolating DNA from 10^6^ *E. histolytica* trophozoites in culture and then serially diluted. The primer and probe sequences were as follows: forward primer Eh-f, AAC AGT AAT AGT TTC TTT GGT TAG TAA AA; reverse primer Eh-r, CTT AGA ATG TCA TTT CTC AAT TCA T; probe Eh-YYT, ATT AGT ACA AAA TGG CCA ATT CAT TCA-dark quencher. Primers and probe were purchased from Integrated Technologies, Coralville, IA.

### Cytokine measurement from cecal tissue and cecal content.

Cecal tissue was homogenized by bead beating with buffer consisting of 1 M HEPES and Halt protease inhibitor cocktail (Thermo Fisher Scientific Inc., Rockford, IL) and then kept on ice for 30 min with buffer containing Triton X-100, HEPES, and Halt protease inhibitor cocktail. The homogenate was then spun at 10,000 × *g* for 10 min, and the supernatant was collected for cytokine protein measurement. Cytokines measured by ELISA (R&D Systems) included IL-4, IL-5, IL-9, IL-23, IL-17, IL-22, IL-25, and TNF-α. Cytokine concentrations were normalized to total protein concentration (bicinchoninic acid [BCA] protein assay; Thermo Fisher).

### Mouse H&E staining.

Tissue in Bouin’s fixative solution was cut into cross sections, paraffin embedded, and then stained with hematoxylin and eosin (H&E) by the Histology Core facility at the University of Virginia. Three readers blinded to the identity of the samples scored epithelial disruption (24). The scale was between 0 and 5. Two different fields were chosen randomly to score from each sample.

### RNA extraction and RT-qPCR.

RNA was extracted from the cecal tissue using the RNeasy minikit (Qiagen, Valencia, CA) according to the manufacturer’s instructions. For reverse transcription, total RNA was transcribed using SuperScript III reverse transcriptase (Invitrogen Life Technologies, Inc., Carlsbad, CA). qPCR was performed on reverse-transcribed cDNA using an iQ SYBR green SuperMix (Bio-Rad Laboratories) in the iCycler iQ System (Bio-Rad Laboratories). The following primers were used in this study: for β-actin, β-actin forward, 5′-AGCCATGTACGTAGCCATCC-3′, and β-actin reverse, 5′-CTCTCAGCTGTGGTGGTGAA-3′; for glyceraldehyde-3-phosphate dehydrogenase (GAPDH), GAPDH forward, 5′-TGCACCACCAACTGCTTAGC-3′, and GAPDH reverse, 5′-GGCATGGACTGTGGTCATGAG-3′; for iNOS, iNOS forward, 5′-CTGGAGGAGCTCCTGCCTCATG-3′, and iNOS reverse, 5′-GCAGCATCCCCTCTGATGGTG-3′. Primers were purchased from Integrated DNA Technologies, Inc., Coralville, IA. Chitinase 3-like 1 primer (RT² quantitative PCR [qPCR] primer assay for mouse Chi3l1) and eosinophil peroxidase primer (Mm_Epx_1_SG QuantiTect primer assay) were purchased from Qiagen (Hilden, Germany). The calculated relative quantity of the cytokine mRNA was normalized to that of glyceraldehyde-3-phosphate dehydrogenase (GAPDH) and β-actin mRNA.

### Isolation of lamina propria cells and flow cytometry.

Single-cell suspensions of lamina propria tissue were isolated as previously described (38). Briefly, cecal tissue was washed in Hanks balanced salt solution (HBSS), 5% fetal calf serum (FCS), 25 mM HEPES buffer. Tissue was then diced and incubated in RPMI containing 0.17 mg/ml Liberase TL (Roche) and 30 μg/ml DNase (Roche) for 40 min at 37°C. Digested tissue pieces were passed through 100-μm and 40-μm cell filters to obtain single-cell suspensions. Isolated lamina propria cells were surface stained for markers Ly6G, CD11b, CD45, CD11c, and Siglec-F according to a general flow cytometry protocol. All samples were run on a Becton, Dickinson LSRFortessa flow cytometry apparatus (BD Bioscience) and analyzed with FlowJo software (TreeStar, Ashland, OR).

### Cytokine treatment and neutralization.

CBA/J mice were injected intraperitoneally with 0.5 μg of recombinant IL-25 each day for a total of 8 doses (4 days before challenge through 4 days after challenge with *E. histolytica*); control mice received PBS. For eosinophil neutralization, rIL-25-treated mice received 40 μg anti-Siglec-F (clone 238047; R&D Systems) or IgG2a isotype (clone 54447; R&D Systems) at day −1 and days 1 and 3 postinfection. Mice were euthanized after 7 days of infection. For TNF-α neutralization, mice were treated with 500 μg of anti-TNF-α monoclonal antibody (MAb) (clone XT3.11; BioXcel) or control rat IgG1 (clone HRPN; BioXcel) intraperitoneally at day −1 and days 1 and 3 postinfection. The mice were euthanized at day 7 postinfection. For flow cytometry, mice were treated with rIL-25 for 4 days prior to challenge and euthanized 24 h postchallenge to isolate lamina propria cells.

### Human colon biopsy specimen immunohistochemistry.

Amebic colitis patients’ colon biopsy specimens were from deidentified patients at the International Center for Diarrheal Disease Research, Bangladesh (ICDDR,B). Human biopsy tissues for the control group were obtained from the Biorepository and Tissue Research Facility at the University of Virginia and confirmed negative for tissue pathology upon histological examination. The Biorepository and Tissue Research Facility of the University of Virginia performed immunohistochemistry staining for IL-25 and TNF-α. Scoring was based on intensity and abundance of IL-25 or TNF-α and was done by three readers blinded to the identity of the samples scored. The staining scale was between 0 and 5. The percentage of the visual field that had intense brown staining within one or two villi was scored. There were two different fields randomly chosen from each sample.

### Statistical analysis.

Student’s *t* test or the Mann-Whitney U nonparametric test was used for comparisons between two groups. *P* values of less than 0.05 were considered significant. Statistical analysis was performed using GraphPad Prism (GraphPad Software, Inc., San Diego, CA). All experiments are representative of at least three independent replicates.
